# Supplementary material for: Application of principal component analysis on temporal evolution of COVID-19
Source: PLoS One. 2021 Dec 2;16(12):e0260899. doi: 10.1371/journal.pone.0260899 (PMC8638895; doi:10.1371/journal.pone.0260899)
Supplement: Attachment — Submitted filename: jwl_rebuttal.docx [file pone.0260899.s001.docx]

**Thank you for your fruitful comments.**

**Reviewer #1:** This is an interesting contribution to the existing literature, but the paper suffers from several shortcomings listed in the following comments.
- The paper should be checked by a native.

Ans: A native person checked the paper.

- A discussion section should be added.

Ans: We added the discussion in the manuscript. Please see the last paragraph of result analysis.

- The introduction should be updated by recent researches.

Ans: The introduction is updated by recent articles. We added some sentences in the third paragraph of Introduction part.

- The novelty and contribution should be clearly bolded.

Ans: The novelty is given in the last paragraph of introduction part.

- The authors should consider some works about Data Analysis that can be applied to model different datasets.

Ans: We add the related articles and update our work. We add new references [14-21].

**Reviewer #2:** Outstanding work. PCA in the context of time series is a reliable method to identify similarities between countries in terms of severity and to anticipate new surges. I would suggest to contrast their findings with similar studies in the discussion and to suggest ways to use their analysis in a practical way to forecast new surges and outbreaks. I suggest the authors consider contrasting the study with one produced by our group: Duarte P, Riveros E. Understanding the cycles of COVID-19 incidence: Principal Component Analysis and interaction of biological and socio-economic factors. Annals of Medicine and Surgery. 2021 Jun 1:102437.

Ans: We added the discussion in the last paragraph of result analysis. We add the reference in [16].

**Reviewer #3:** I failed to identify some points as follows:
1. What is the hypothesis and problem that you address in this paper?

**Ans:** We analyze the time series of COVID-19 to extract information about relations and the dynamics of COVID-19 in the top 25 countries. We want to investigate the dynamical similarity among countries. The similarity of strategic policies should be influenced to control the COVID-19. We gave some points in last paragraph of Introduction.

2. Does only PCA enough to explain this complex issue as the scientist are striving to know the behavior of the virus and its mutation.

**Ans:** Of course, only PCA is not enough to explain these issues. However, we believe that the PCA is one of the powerful methods to understand the dynamics of disease spreading. Genome sequence analysis is necessary to know the behavior of the virus and its mutation. However, genome sequence analysis is critical in understanding the COVID-19 and preventing the spread of the disease. Different techniques such as PCA, Machine learning, artificial neural network can be applied to analyze genome sequence of COVID-19. We will apply these techniques on gene data for further investigation. We include some methods to explain the dynamical features of COVID-19 in second paragraph of Introduction part.

3. What could be the implications of your study in epidemiology?

**Ans:** The transmission of diseases in the severe affected countries can be understood by using PCA. From the PCA, we can observe the strong correlation of the dynamical change for the spreading disease. We can propose the similar strategic policies to those countries. I include these points in last paragraph in Conclusion part

4. Is that only focus to check the applicability of PCA methods?

**Ans:** Actually, we apply PCA technique to the correlations for the change of COVID-19 cases and death among the severe affected countries to understand the states and dynamic of COVID-19 in the analyzed countries. This analysis is also helpful to determine the relationships among these countries. In future, we will apply feature ranking of machine learning on gene data to understand the behavior of virus and its mutation.

5. `By studying the temporal evolution
of the correlations of the different affected countries, we uncover the periods when there were major
changes in the correlation structure of the global pandemic in the analyzed countries.` to know this country wise data is enough I think what new things can be added by this research? is unclear.

**Ans:** PCA is a technique to reduce data dimension. Applying PCA on correlation structure, we try to show that only first two PCs are enough to observe the changes of the states in the analyzed countries and to make relationships among these countries. We gave some comments in the last paragraph in section <Pandemic states of countries>.

6. The variation in confirmed cases and deaths among different countries is obvious it need not to say after PCA.

Ans: Yes. You are right. We wanted to identify the periods to observe the COVID-19 states in the analyzed countries by heat map so that we can make a comparative study among the countries in the basis of severity.

7. Why the dynamic trend is changing is not explored here. Multiple factors might be in fact certainly involved from governance to economy.

Ans: The change of correlation structure among the countries is identified by the largest eigenvalue and is used to observe the dynamics of COVID-19. The correlation structure for the change of COVID-19 cases and deaths among the countries is altered due to lock down, making social distance, hard immunity and for other policies and consequently, the dynamic trend is changed. Some articles considered other factors like socio-economic factors in new added Ref [16]. In the future study, we will consider other possible factors.
